# Comparative analysis of potential broad-spectrum neuronal Cre drivers

**DOI:** 10.12688/wellcomeopenres.17965.1

**Published:** 2022-07-08

**Authors:** Katie M Paton, Jim Selfridge, Jacky Guy, Adrian Bird

**Affiliations:** 1 Wellcome Centre for Cell Biology, University of Edinburgh, Edinburgh, EH9 3BF, UK

**Keywords:** Cre driver mice, Cre-Lox technology, neurodevelopment, Synapsin1, Snap25, MeCP2 reporter

## Abstract

Cre/Lox technology is a powerful tool in the mouse genetics tool-box as it enables tissue-specific and inducible mutagenesis of specific gene loci. Correct interpretation of phenotypes depends upon knowledge of the Cre expression pattern in the chosen mouse driver line to ensure that appropriate cell types are targeted. For studies of the brain and neurological disease a pan-neuronal promoter that reliably drives efficient neuron-specific transgene expression would be valuable. Here we compare a widely used “pan-neuronal” mouse Cre driver line, *Syn1-cre*, with a little-known alternative, *Snap25-IRES2-cre*. Our results show that the *Syn1-cre* line broadly expresses in the brain but is indetectable in more than half of all neurons and weakly active in testes. In contrast the *Snap25-IRES2-cre* line expressed Cre in a high proportion of neurons (~85%) and was indetectable in all non-brain tissues that were analysed, including testes. Our findings suggest that for many purposes *Snap25-IRES2-cre* is superior to *Syn1-cre* as a potential pan-neuronal cre driver.

## Introduction

Cre/Lox technology has been used extensively in mice to conditionally knock out or reactivate a gene of interest in a specific cell lineage or at a particular developmental stage. This has led to the generation of many transgenic Cre driver lines that specifically express the *cre* recombinase gene in a variety of cell types. Here we focus on Cre drivers that are potentially expressed in neurons across a broad range of subtypes, as these would be valuable tools for studying brain-wide molecular mechanisms and neurological disease. We compared two ‘pan-neuronal’ transgenic lines, *Syn1-cre* and *Snap25-IRES2-cre*, that are reported to drive Cre expression in neurons only ( Harris *et al.*, 2014; Zhu *et al*., 2001). *Syn1* and *Snap25* gene promoters are good candidates to drive pan-neuronal gene expression as both encode proteins with functions common to most neurons. Synapsin-1 modulates neurotransmitter release at pre-synaptic nerve terminals by tethering synaptic vesicles to actin filaments in a phosphorylation-dependent manner (reviewed by Cesca *et al.*, 2010). Its expression is widespread throughout the central and peripheral nervous system but is not apparent in non-neuronal cells (reviewed by De Camilli *et al.*, 1990). Synaptosomal-Associated Protein, 25kDa (SNAP-25) is a component of the SNARE ( Soluble *N* -ethylmaleimide-sensitive factor Attachment protein Receptor) complexes which mediate synaptic vesicle docking and fusion during exocytosis ( Rizo & Südhof, 2002). SNAP-25 is expressed almost exclusively in the brain, whereas its ortholog SNAP-23, which is ~60% identical to SNAP-25, is expressed ubiquitously ( Ranichandran *et al.*, 1996; Wang *et al.*, 1997).

The *Syn1-cre* transgenic line developed by Zhu *et al*. (2001) has been described in the literature as a ‘pan-neuronal’ Cre driver and has been used in many studies for neuron-specific deletion of a Cre-inducible gene (Mouse Genome database: www.informatics.jax.org/allele/key/6193). Cre recombinase is expressed from a 4.3 kb fragment of the rat Synapsin-1 gene ( *Syn1*) promoter ( Hoesche *et al*., 1993). The second “pan-neuronal” Cre driver line, *Snap25-IRES2-cre*, was generated by the Allen Institute for Brain Science and is described as the having “strong widespread expression throughout the brain” ( connectivity.brain-map.org/transgenic) ( Harris *et al.*, 2014). The *Snap25-IRES2-cre* allele is a *cre* knock-in allele in which an Internal Ribosome Entry site (IRES) and the *cre* recombinase gene sequence are inserted downstream of the stop codon of the endogenous *Snap25* gene ( www.informatics.jax.org/allele/MGI:5507846). Here we compare the Cre expression pattern of these two driver lines using a ubiquitously expressed fluorescent *Mecp2 ^mCherryStop^* Cre reporter line. We address the Cre recombination efficiencies, across the brain, specifically in neurons, and in a panel of non-neuronal tissues. Our findings suggest that *Snap25-IRES2-cre* drives Cre expression in a more brain-specific manner and in a far higher fraction of all neurons than *Syn1-cre*. We further discuss the limitations of our characterisation, in particular developmental trajectory and remaining uncertainty regarding Cre expression by both lines in glia.

## Methods

### Mice

All experiments involving mice were approved by the local Animal Welfare Ethical Review Body (AWERB) of University of Edinburgh and were part of a project licence (PPL P3C15137E) approved by the the Home Office, UK, under the Animals (Scientific Procedures) Act 1986. Animals were housed in individually ventilated cages along with a housing dome, nestlet, chew stick and tube/tunnel for environmental enrichment. B6;129S- *Snap25 ^tm2.1(cre)Hze^*/J (RRID:IMSR_JAX:023525), B6.Cg-Tg(Syn1-cre)671Jxm/J (RRID:IMSR_JAX:003966) and B6.C-Tg(CMV-cre)1Cgn/J (RRID:IMSR_JAX:006054) mice were acquired from the Jackson Laboratory and bred and maintained on a C57BL/6JCrl background. The *Mecp2* Cre reporter line: *Mecp2 ^mCherryStop^* was generated by gene targeting in ES cells followed by blastocyst injection as described ( Guy *et al*., 2007). The targeting vector used was a modified version of the *Mecp2 ^lox-Stop^* targeting vector ( Guy *et al*., 2007), with the addition of a C-terminal mCherry tag. *Mecp2 ^mCherryStop^* mice were crossed to B6.C-Tg(CMV-cre)1Cgn/J mice to achieve the *Mecp2 ^mcherry^* mice, constitutively expressing MeCP2-mCherry. The *Mecp2 ^mCherryStop^* and *Mecp2 ^mcherry^* mice were maintained on a C57BL/6JCrl background. Male mice hemizygous for the modified X-linked Mecp2 allele were used for analysis to ensure constitutive expression of the allele as opposed to mosaic expression which occurs in heterozygous females. Tissues were harvested from 6–8-week-old mice, flash frozen in liquid nitrogen and stored at −80°C before processing for protein, nuclei or DNA. All efforts were made to ameliorate any suffering: *Mecp2 ^mCherryStop/y^* mice were sacrificed before the onset of neurological symptoms and decline in health status to prevent suffering. For each experiment the minimum number of mice (2–3 of each genotype) were used to provide meaningful data where descriptive statistics (i.e mean and standard deviation) could be performed.

### Western blot analysis

Whole-cell protein was extracted from tissues by homogenizing tissues using an Ultra-Turrax T25 homogeniser in NE1 buffer (20 mM HEPES pH 7.9, 10 mM KCL, 1 mM MgCl _2_, 0.1% Triton X-100, 20% glycerol, 0.5 mM DTT, protease Inhibitors cocktail (Roche)) followed by incubation with 1000 U/mL Benzonase (Sigma E1014) for 15 minutes at room temperature. An equal volume of 2× Laemmli Sample Buffer (Sigma S3401) was added and samples were boiled for 5 minutes, snap frozen on dry ice, boiled again before centrifuging (16,100×g, 5 minutes, 4°C), taking the supernatant. For Western analysis protein samples were run on Bio-Rad TGX (4–20%) gradient gels and blotted onto 0.2 μM nitrocellulose membrane by overnight wet transfer at 25 V and 4°C in 25 mM Tris, 192 mM glycine. Membranes were blocked with 5% skimmed milk powder in TBS + 0.1% Tween-20 and incubated overnight at 4°C with primary antibodies diluted in the same blocking solution (1:5000 Anti-mCherry rabbit polyclonal (Abcam ab167453) and 1:10,000 Anti-Histone H3 rabbit polyclonal (Abcam ab1791)). After washing in TBS + 0.1% Tween-20, membranes were incubated for 1–3 hours at room temperature with IRDye donkey anti-rabbit 680LT secondary antibody diluted (1:10,000 LICOR Biosciences 926-68023). Membranes were imaged on the LI-COR® Odyssey CLx imaging system. Images were quantified using Image Studio Lite software (LI-COR biosciences); three biological replicates for each genotype were analysed.

### Flow cytometry

Brain tissue was gently homogenized using loose dounce homogenisers in 5 mL ice-cold homogenization buffer (HB) (320 mM sucrose *,* 5 mM CaCl, 3 mM MgAc, 10 mM Tris pH 8.0, 0.1 nM EDTA, 0.1% NP40, 0.1 mM PMSF, 1 mM B-Mercaptoethanol, 1× Protease inhibitors [Roche]) and 5 mL of 50% OptiPrep gradient centrifugation medium (50% OptiPrep [Sigma D1556], 5 mM CaCl, 3 mM MgAc, 10 mM Tris-HCl pH 8.0, 0.1 mM PMSF, 1 mM β-mercaptoethanol in dH _2_O) was added. Lysate plus 50% OptiPrep was loaded onto 10 mL 29% iso-osmolar OptiPrep solution (29% OptiPrep in dH _2_O) in Beckman Coulter Ultra clear Ultracentrifugation tubes and samples were centrifuged at 10,100×g for 30 minutes at 4°C. Nuclei were resuspended in 2 mL of 5% glycerol, 1× protease inhibitors in PBS, flash frozen in liquid nitrogen and stored at −80°C. For flow cytometry, nuclei were pelleted at 600×g for 5 minutes at 4°C, washed in 500 µL of PBTB (2.5 g bovine serum albumin [Sigma A9418], 0.1% Triton, 1× protease inhibitors in PBS filter sterilized) and resuspended in 250 µL PBTB. NeuN primary antibody (Millipore MAB377) conjugated to Alexa Fluor ^TM^ 647 (APEX ^TM^ Alexa Fluor ^TM ^Antibody labelling Kit) was added at a dilution of 1:125 and incubated for 45 minutes at 4°C on a rotating wheel protected from light. Labelled nuclei were analysed by flow cytometry directly following antibody staining.

Flow cytometry (LSRFortessa SORP flow cytometer [Beckton Dickinson Immunocytometry Systems] using BD FACSDiva v8 Software) was used to acquire fluorescence measurements for approximately 50,000 single nuclei per sample. MeCP2 ^mCherry^ reporter protein was detected following 561 nm excitation at emission between 610/20 nm and Alexa Fluor ^TM^ 647 conjugated anti-NeuN (NeuN-AF647) was detected following 640 nm excitation at emission between 660/20 nm. Compensation was performed to avoid spectral overlap. Flow cytometry data was further analysed using FlowJo version 10.8.1. Intact nuclei were gated by forward scatter (FSC-A) and side scatter (SSC-A) plots. Singlets were then gated according to FSC-H *versus* FSC-A plots. The NeuN-high population were gating according to cell density using FSC-A *versus* NeuN-AF647 fluorescence (this was done manually for each replicate as the plots shifted slightly between replicates). Using the *Mecp2 ^mcherry/y^* positive and *Mecp2 ^mcherrystop/y^* negative controls, the mCherry-high and mCherry-low populations were gated to quantify the percentages of nuclei in the NeuN-high and NeuN-low populations which fall into the mCherry-high and mCherry-low gates. The full gating strategy is shown in Supplementary figure 1 in the extended data ( Paton *et al.,* 2022).

### Immunofluorescence

Mice were perfused with 4% paraformaldehyde (Sigma 158127) in PBS, pH 7.4 before dissection of the brain tissue. Dissected brains were immersion fixed in PFA overnight at 4°C. The brain tissue was then dehydrated in 30% sucrose in PBS for 24 hours, before being washed in PBS and flash frozen in isopentane cooled on dry ice. Brains were sectioned into 10-μM sections using a Cryostat (Leica CM1900) and mounted onto Superfrost Plus microscope slides (VWR 631-0108). For immunofluorescence, brain sections were washed in PBS, permeabilised with 0.1% Triton X-100 in PBS for 15 minutes, and washed again. Sections were then blocked in 1.5% normal goat serum for 60 minutes, incubated overnight at 4°C with conjugated Anti-NeuN-fitc488 antibody (Millipore MAB377X) diluted 1:200 in blocking buffer, washed, incubated for 10 minutes in 1 μg/mL DAPI in PBS, and washed again. Finally, sections were mounted in ProLongDiamond (Life Technologies P36961), allowed to set overnight in the dark at room temperature before visualising sections using LeicaSP5 Confocal Laser Scanning Microscope.

### Southern blot analysis

Brain tissue (one hemisphere) was homogenised using a loose Dounce homogeniser in 3 mL lysis buffer (50 mM Tris.Cl pH 7.5, 100 mM NaCl, 5 mM EDTA) to form a uniform suspension and 1% SDS (Sigma L4509) and 4 mg/mL Proteinase K (Bioline BIO-37085) were added followed by incubation overnight at 55°C. Homogenates were incubated for a further 1–2 hours at 37°C with 0.1 mg/mL RNaseA (Thermo Scientific EN0531). DNA was extracted with an equal volume of phenyl:chloroform:isoamyl alcohol (Sigma P3803 Invitrogen 15593-031) and centrifuged (15 minutes 2095×g). DNA was precipitated from the aqueous phase using 0.1 v NaOAc (Sigma S7899) and 2.5 v ~100% EtOH. DNA was rinsed in 80% EtOH and dissolved in TE. For Southern blot analysis, genomic DNA (10 µg per lane) was digested with 3 μL of EcoR1-HF (NEB R3101) restriction enzyme for around 8 hours at 37°C. Digested DNA was run on 0.8% agarose gel containing EtBr at 36V for ~18 hours. Gels were incubated in 0.25 M HCl for 15 minutes and 0.4 M NaOH for 45 minutes before being transferred to Zeta-Probe GT (Bio-Rad 1620159) or AmershamTM Hybond N+ Nylon Blotting membrane (GE Healthcare RPN303 S) by dry transfer. Membranes were washed in 2× SSC (300 mM NaCl, 30 mM Na citrate) before being blocked with modified Church & Gilbert hybridisation buffer (0.5 M NaPi, 7% SDS, 1 mM EDTA, 10 mg/mL BSA), 50 μg/mL herring sperm ssDNA (Sigma D7290) at 65°C. Membranes were hybridised overnight at 65°C with a radiolabelled 1.2-kb NcoI–BamHI fragment of the 3′UTR of *Mecp2* ( Guy *et al.*, 2001)). The Prime-a-Gene Labelling System (Promega U1100) was used to label the probe with [α32]dCTP. Membranes were washed briefly with room temperature 2× SSC (20X SSC: 24.8 mM Tris, 192 mM glycine,1% (w/v) SDS), followed by two 20-minute washes in 2× SSC + 1% SDS at 65°C before being visualised by phosphorimager (Typhoon FLA 7000). 

## Results

**Figure 1.**
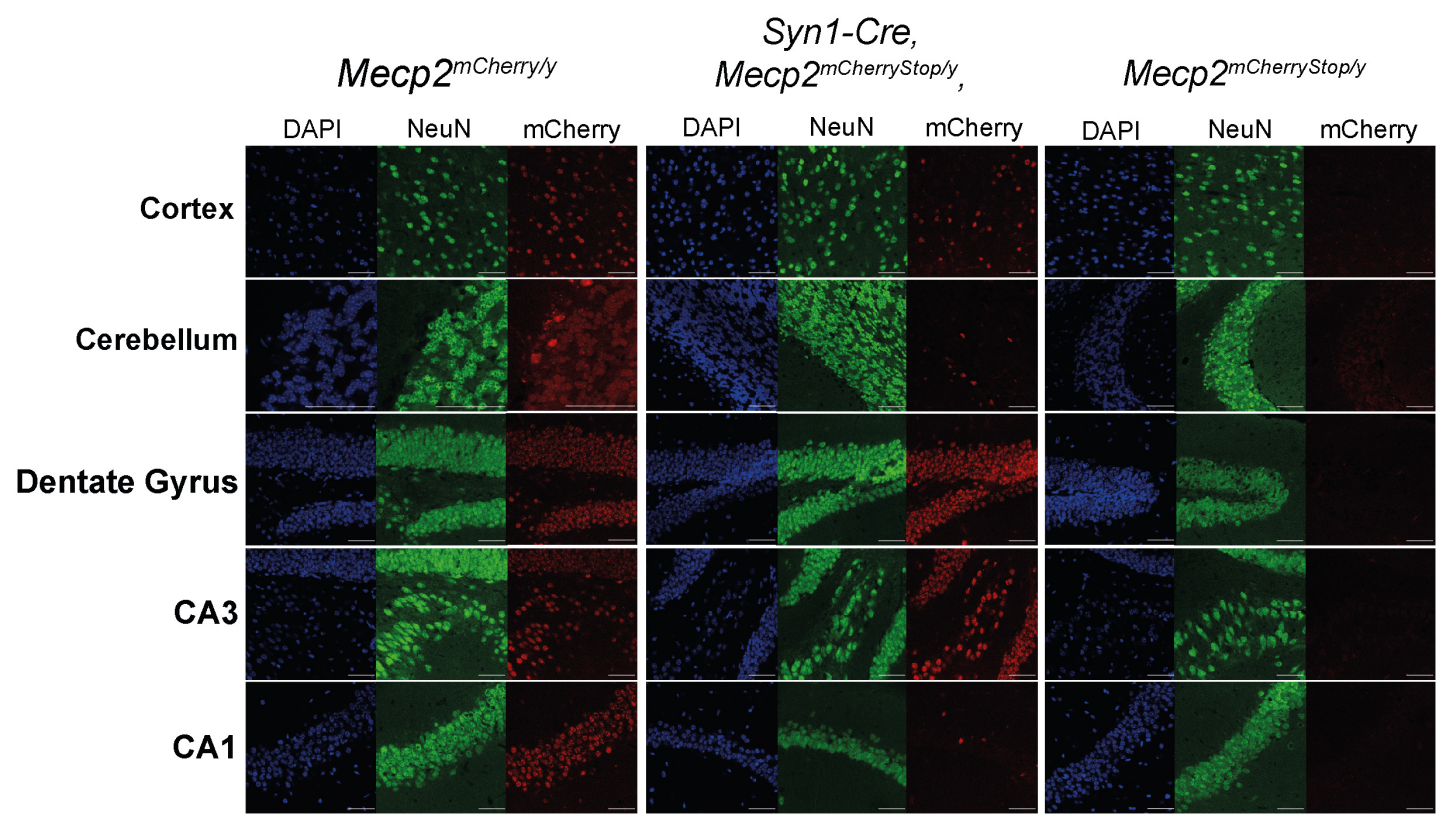
*Syn1-cre* expression is variable across different brain regions. The Cre-mediated activation of MeCP2 ^mCherry^ fluorescence was visualised in brain sections from *Syn1-cre,Mecp2 ^mCherryStop/y^* mice, and *Mecp2 ^mCherryStop/y^* and *Mecp2 ^mCherry/y^* control mice. Images shown are taken from one mouse per genotype. All mice used were male *Mecp2* hemizygotes. Sections were stained for DAPI and immunolabelled for the neuronal marker NeuN. Scale bars: 50 µm.

To test for brain-wide Cre expression, we analysed Cre activity in *Syn1-cre* mice using a mouse reporter line in which the endogenous *Mecp2* gene was tagged by fusion to an mCherry reporter and silenced by insertion of a transcriptional *Stop* cassette into intron 2. Cre-mediated excision of the *Stop* cassette leads to robust expression of MeCP2 ^mCherry^ which is visualised as characteristic punctate nuclear staining ( Nan *et al.*, 1996). The *Mecp2 ^mCherry^* mice which constitutively express MeCP2 ^mCherry^ in all somatic cell types were used as a positive control. These mice were generated by crossing the *Mecp2 ^mCherryStop^* line to a *CMV-cre* line which has ubiquitous Cre activity. Comparing brain mCherry expression between *Syn1-cre* and *Mecp2 ^mCherry^* control mice by fluorescence microscopy showed incomplete deletion of the *Stop* cassette in the cortex, efficient deletion in the dentate gyrus and CA3 region, but MeCP2 activation in the cerebellum and CA1 region of the hippocampus was minimal ( Figure 1). Zhu and colleagues (2001) also showed low Cre activity in the cerebellum of *Syn1-cre* mice using a transgenic *loxP-Stop LacZ* Cre-reporter strain. Whole brain *in situ* hybridisation data from the Allen Brain Institute also indicated patchy expression of *Syn1-cre* in multiple regions of the mouse brain. In conclusion the *Syn1-cre* line gives uneven Cre expression and cannot be considered as a “pan-neuronal” Cre diver.

Seeking a more global driver of Cre expression in the brain, we turned to the publicly available Transgenic Characterization database of the Allen Brain Institute ( connectivity.brain-map.org/transgenic), which includes anatomical data from over 100 Cre driver lines. From this database we identified the *Snap25-IRES2-cre* line, which reportedly exhibited “strong widespread expression throughout the brain” ( connectivity.brain-map.org/transgenic) ( Harris *et al.*, 2014). Cre recombinase is bicistronically expressed with endogenous *Snap25* so its expression pattern should closely follow the pattern of endogenous *Snap25* mRNA. Despite its promising properties, the *Snap25-IRES2-cre* line is less well characterised than the *Syn1-cre* line and has been little used.

**Figure 2.**
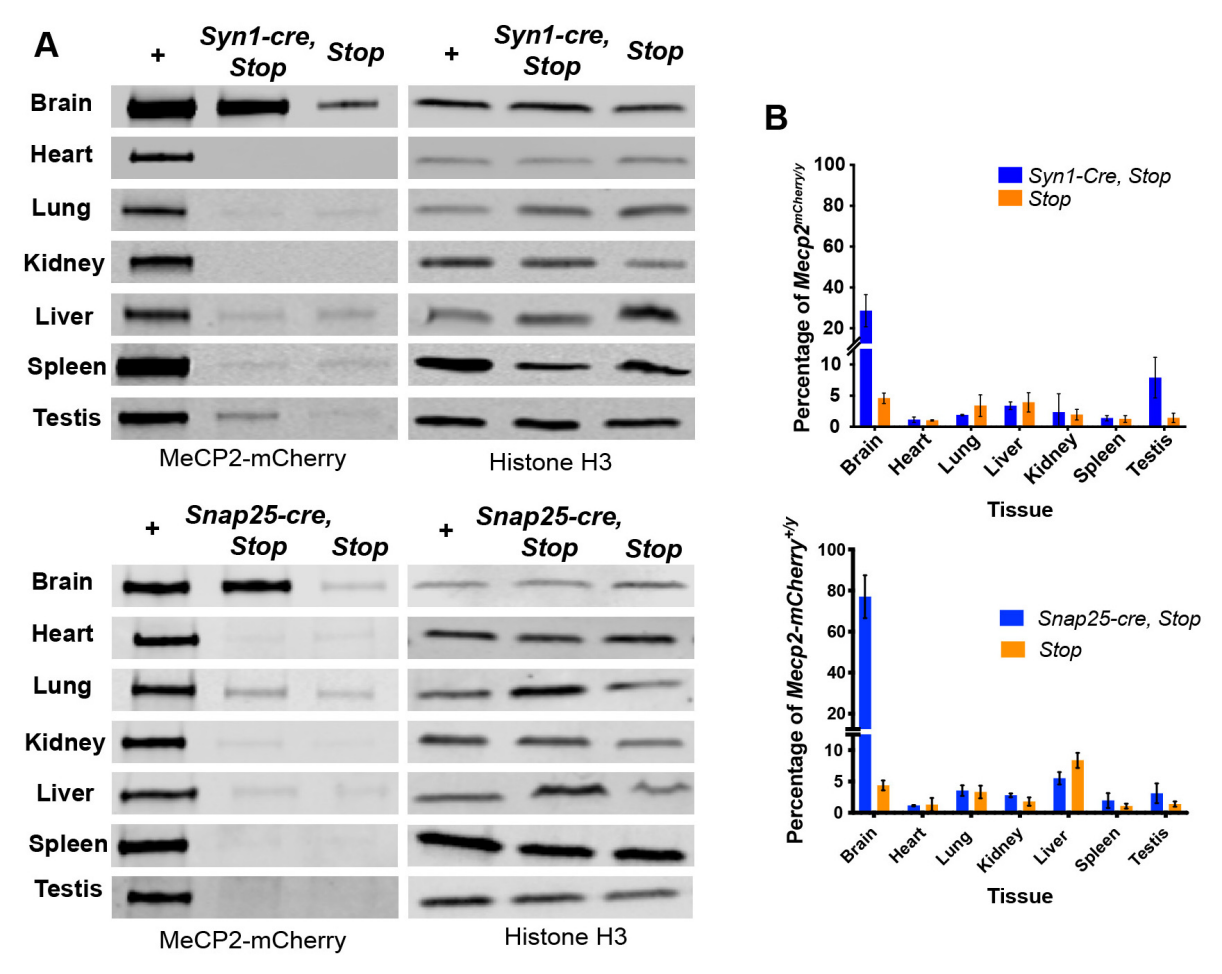
Cre recombinase expression in *Syn1-cre* and *Snap25-IRES2-cre* transgenic mice is restricted to the brain. **A**) Representative images of western blots of MeCP2 ^mCherry^ expression in a panel of different tissues from: constitutively expressing *Mecp2 ^mCherry/y^* (+) and *Mecp2 ^mCherryStop/y ^(Stop)* control mice, and *Syn1-cre,Mecp2 ^mCherryStop/y^* ( *Syn1-cre,Stop*) mice or *Snap25-IRES2-cre,Mecp2 ^mCherryStop/y^* ( *Snap25-cre,Stop*) mice. **B**) Graphs showing the MeCP2 ^mCherry^ protein level in *Stop* and *Syn1-cre,Stop* or *Snap25-cre,Stop* tissues as a percentage of the level in constitutively expressing *Mecp2 ^mCherry/y^* (+). Three biological replicates (3 separate mice) per genotype, per tissue were analysed. Western blot quantification was performed using Image Studio Lite Software (LI-COR biosciences). The MeCP2 ^mCherry^ signal intensity was normalised to the signal intensity of the H3 loading control for each sample. Plotted here is the mean and standard deviation for the normalised protein level for each genotype as a percentage of the average of the constitutively expressed *Mecp2 ^mCherry/y^* (+) samples.

To compare expression and activity of Cre between these driver lines, and using the constitutive *Mecp2 ^mCherry^* line as a control, we determined the expression of MeCP2 ^mCherry^ in brain by semi-quantitative western blotting ( Figure 2). The low level of MeCP2 ^mCherry^ expression in tissues seen in the *Mecp2 ^mCherryStop/y^* negative control is due to leaky expression of the *Stopped* allele that has been previously reported ( Guy *et al.*, 2007; Robinson *et al.*, 2012), rather than Cre mediated removal of the *Stop* cassette. This assay indicated that *Snap25-IRES2-cre* restored brain MeCP2 ^mCherry^ expression to ~80% of the wildtype level, whereas *Syn1-cre* yielded only ~30% restoration. The majority of MeCP2 ^mCherry^ protein in whole brain protein extracts is derived from neurons which express on average at least six-fold more MeCP2 per cell than glia ( Skene *et al.*, 2010). Accounting for this difference, the relatively low MeCP2 ^mCherry^ level in the *Syn1-cre,Mecp2 ^mCherryStop/y^* brain agrees with the patchy distribution of Cre activity in neurons according to immunofluorescence ( Figure 1). Analysis of mouse tissues other than brain revealed minimal Cre activity. The level of MeCP2 ^mCherry^ expression in *Syn1-cre,Mecp2 ^mCherryStop/y^* and *Snap25-IRES2-cre,Mecp2 ^mCherryStop/y^* mice was similar to that of *Mecp2 ^mCherryStop/y^* control mice, suggesting that Cre-mediated deletion of the *Stop* cassette had not occurred. The only exception was a low level of MeCP2 ^mCherry^ activation detected in testes of *Syn1-cre* mice, in line with previous reports ( Luo *et al.*, 2020; Rempe *et al.*, 2006). No evidence of cre activity in testes was seen in the *Snap25-IRES2-cre* line. These experiments demonstrate that activity of both Cre drivers is largely confined to the brain, but that the *Snap25-IRES2-cre* line is a more effective driver of Cre in the brain than *Syn1-cre*.

**Figure 3.**
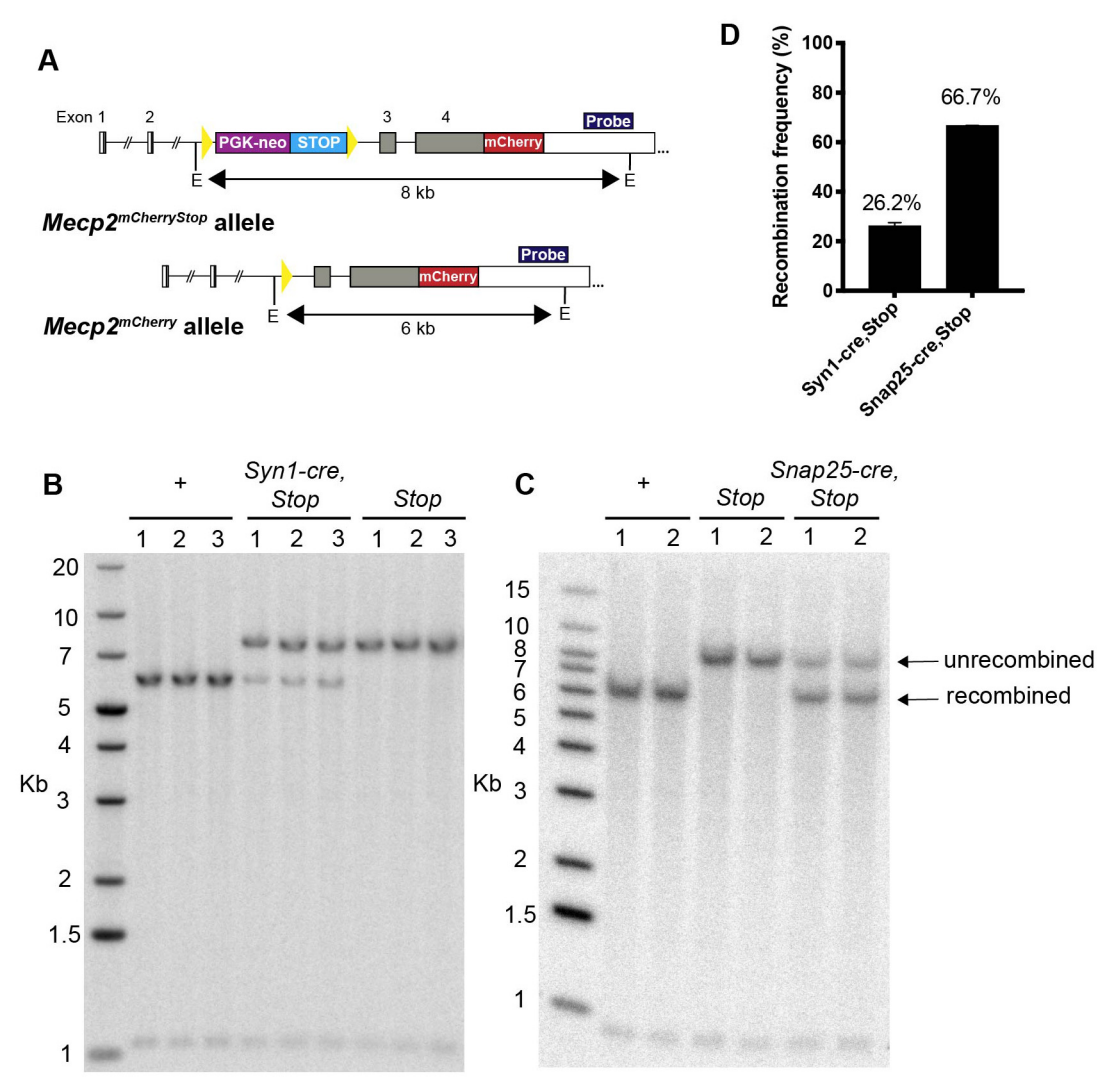
Cre-mediated recombination frequency is substantially higher in *Snap25-IRES-cre,Mecp2 ^mCherryStop/y^* compared to *Syn1-cre,Mecp2 ^mCherryStop/y^* mouse brain. **A**) Southern blot screening strategy for identifying the recombined *Mecp2 ^mCherry^* and unrecombined *Mecp2 ^mCherryStop^* allele. The position of the 3’UTR hybridisation probe is indicated in dark blue and E indicates the position of the EcoRI restriction enzyme cut sites. **B**) and **C**) Southern blot of genomic DNA extracted from whole brain tissue of *Mecp2 ^mCherry/y^* (+) and *Mecp2 ^mCherryStop/y^* ( *Stop*) control mice, and *Syn1-cre,Mecp2 ^mCherryStop/y^* ( *Syn1-cre, Stop*) mice or *Snap25-IRES2-cre,Mecp2 ^mCherryStop/y^* ( *Snap25-cre, Stop*) mice. Each lane on the Southern blot corresponds to a sample from a separate mouse (n=2 or 3 of each genotype). **D**) Histogram showing the mean and standard deviation of the recombination frequency quantified from the Southern blots in **B**. Quantification of the signal intensity of each band was done using ImageJ-Fiji and the intensity of the recombined band was expressed as a percentage of the total of both recombined and unrecombined bands.

To quantify the total recombination frequency throughout the brain, Southern blot analysis was performed on genomic DNA preparations from whole brain samples for *Mecp2 ^mCherryStop/y^, Mecp2 ^mCherry/y^, Syn1-cre,Mecp2 ^mCherryStop/y^* and *Snap25-IRES2-cre,Mecp2 ^mCherryStop/y^* mice ( Figure 3). The DNA was digested with EcoRI and the *Mecp2* locus visualised using a radiolabelled probe which annealed to the 3′UTR to distinguish an 8 kb fragment derived from the unrecombined locus in *Mecp2 ^mCherryStop/y^* from the 6 kb fragment produced from the recombined locus. Quantification of unrecombined and recombined fragments showed a recombination frequency of 26.2% in *Syn1-cre,Mecp2 ^mCherryStop/y^* mice compared with 66.7% in *Snap25-IRES2-cre,Mecp2 ^mCherryStop/^* ^y ^mice ( Figure 3). By comparison, *Nes-cre,* which drives expression in both neurons and glia, leads to >90% recombination of the *Mecp2 ^Stop^* allele in mouse brain ( Ross *et al.*, 2016). Southern blot analysis of whole brain extracts does not allow the recombination frequency in neurons and non-neuronal cells to be distinguished, but it is of interest that recent estimates suggest around 65% of all the cells in the mouse brain are neurons ( Erö *et al.*, 2018; Herculano-Houzel *et al.*, 2006; Herculano-Houzel, 2014) (Blue Brain Cell Atlas: bbp.epfl.ch/nexus/cell-atlas/) which is close to the recombination frequency in the *Snap25-IRES2-cre,Mecp2 ^mCherryStop/y^* brain. In contrast, the recombination frequency in *Syn1-cre,Mecp2 ^mCherryStop/y^* brains suggests that less than half of all neurons have undergone recombination.

**Figure 4.**
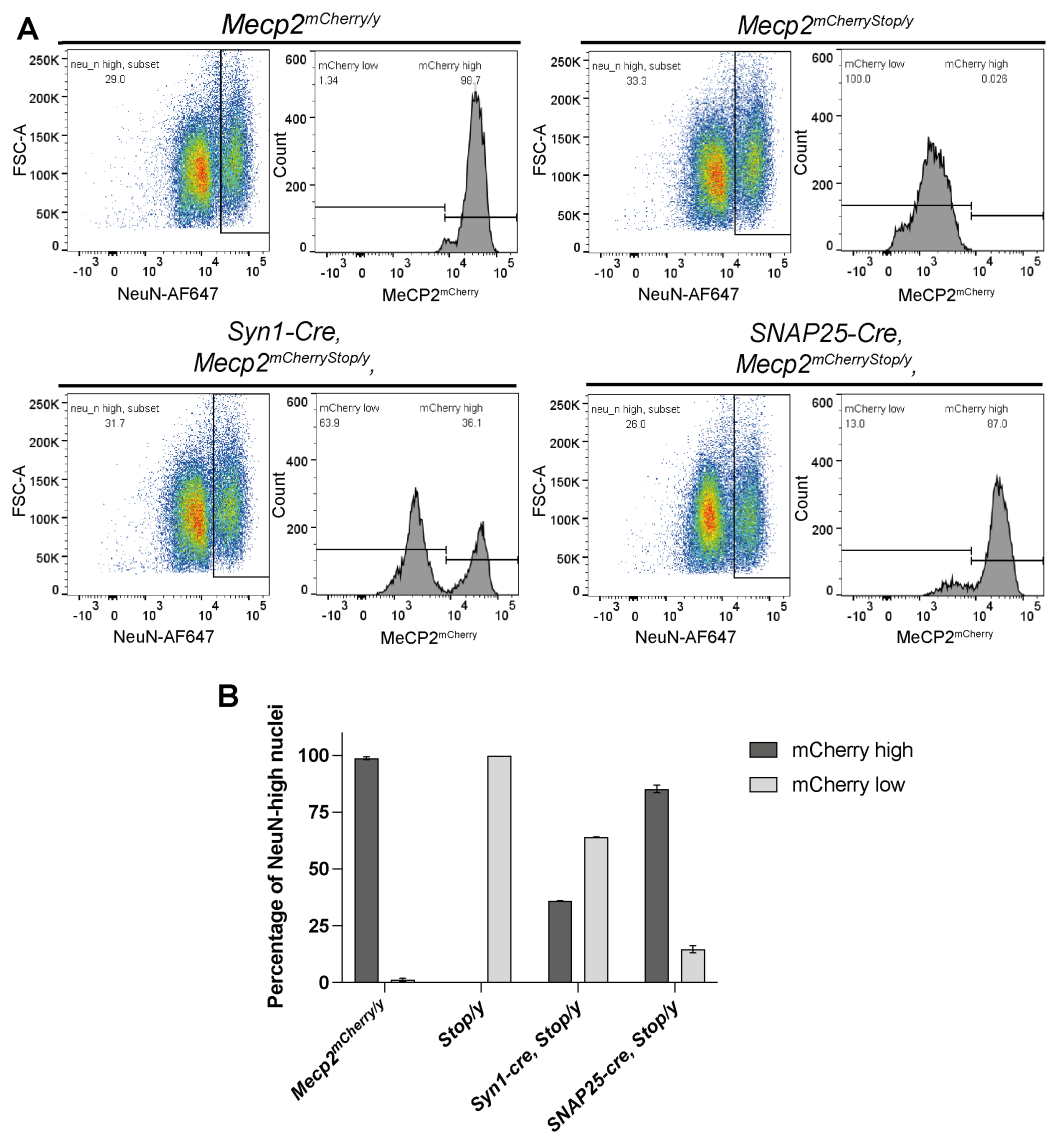
*Snap25-IRES2-cre* is superior to *Syn1-cre* as a pan-neuronal Cre driver. **A**) Flow cytometry analysis of nuclei extracted from *Mecp2 ^mCherry/y^, Mecp2 ^mCherryStop/y^ (Stop/y), Syn1-Cre,Mecp2 ^mCherryStop/y^*, *Snap25-IRES2-Cre,Mecp2 ^mCherryStop/y^* ( *Snap25-cre,Mecp2 ^mCherryStop/y^*) brains. Shown here are representative plots for each genotype showing the gating strategy used to identify the proportion of NeuN-high neuronal nuclei which have activated MeCP2 ^mCherry^ expression by flow cytometry. Intact, single nuclei (gating strategy shown in the Supplementary figure 1) were gated and analysed for their NeuN expression (plot 1: y axis: Forward Scatter FCS-A; x axis: NeuN-APC647 fluorescence). The NeuN-high populations were then gated and analysed for MeCP2 ^mCherry^ expression (plot 2: y axis: nuclei count; x axis: mCherry/PE-Texas Red fluorescence.) **B**) Bar charts showing the proportion of NeuN-high nuclei which are within the MeCP2 ^mCherry^-high (dark grey) or MeCP2 ^mCherry^-low (light grey) gate for three biological replicates of each genotype. Plotted is the mean and standard deviation.

To distinguish the Cre reporter expression patterns in neurons as opposed to all brain cells, we analysed brain nuclei based on mCherry fluorescence combined with immunostaining for NeuN, a nuclear protein expressed in the majority of neurons but absent in glia ( Mullen *et al.*, 1992). Nuclei from *Syn1-cre,Mecp2 ^mCherryStop/y^* and *Snap25-IRES2-cre,Mecp2 ^mCherryStop/y^* were stained with fluorescently labelled NeuN and analysed by flow cytometry to determine what proportion of NeuN expressing cells were expressing MeCP2 ^mCherry^ ( Figure 4). Nuclei from *Mecp2 ^mCherryStop/y^* and *Mecp2 ^mCherry/y^* mouse brains served as negative and positive controls respectively. Approximately 50,000 single nuclei were gated to give NeuN-high and NeuN-low populations. The NeuN-high population in *Mecp2 ^mCherry/y^* control samples displayed a single population of nuclei highly expressing mCherry that was absent in the *Mecp2 ^mCherryStop/y^* negative control samples ( Figure 4). Focussing only on NeuN-high neuronal population, recombination leading to reactivation of the *Mecp2 ^mCherryStop^* allele occurred in 36% of NeuN-high nuclei (neurons) for *Syn1-cre* and 85% for *Snap25-IRES2-cre*, suggesting that the *Snap25-IRES2-cre* is far superior in terms of pan-neuronal Cre expression.

**Figure 5.**
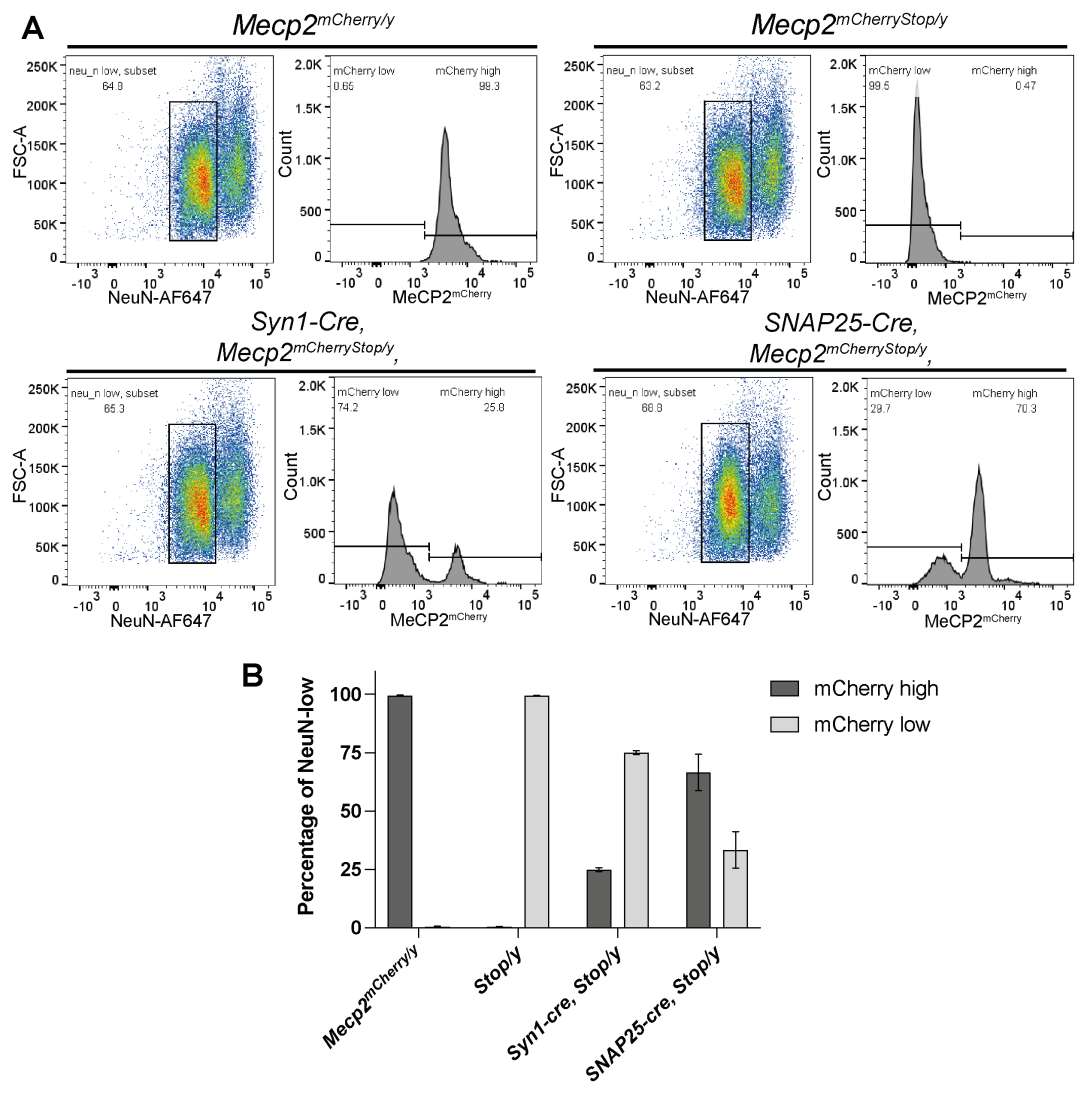
Flow cytometry analysis revealed substantial amounts of Cre recombination in both *Snap25-IRES2-cre* and *Syn1-cre* NeuN-low brain nuclei. **A**) Flow cytometry analysis of nuclei extracted from *Mecp2 ^mCherry/y^, Mecp2 ^mCherryStop/y^ (Stop/y), Syn1-Cre,Mecp2 ^mCherryStop/y^*, *Snap25-IRES2-Cre,Mecp2 ^mCherryStop/y^* ( *Snap25-cre,Mecp2 ^mCherryStop/y^*) brains. Shown here are representative plots for each genotype showing the gating strategy used to identify the proportion of NeuN-low neuronal nuclei which have activated MeCP2 ^mCherry^ expression by flow cytometry. Intact, single nuclei (gating strategy shown in the Supplementary figure 1) were gated and analysed for their NeuN expression (plot 1: y axis: Forward Scatter FCS-A; x axis: NeuN-APC647 fluorescence). The NeuN-low populations were then gated and analysed for MeCP2 ^mCherry^ expression (plot 2: y axis: nuclei count; x axis: mCherry/PE-Texas Red fluorescence.) **B**) Bar charts showing the proportion of NeuN-low nuclei which are within the MeCP2 ^mCherry^-high (dark grey) or MeCP2 ^mCherry^-low (light grey) gate for three biological replicates of each genotype. Plotted is the mean and standard deviation.

While the NeuN-high peak is likely to contain only neuronal nuclei ( Herculano-Houzel & Lent, 2005), the content of NeuN-low peaks is less easy to define ( Figure 5). Glial cells are NeuN-negative, but also some neuronal subtypes, including cerebellar Purkinje cells, olfactory bulb mitral cells and retinal photoreceptor cells, are reportedly devoid of NeuN immunoreactivity ( Mullen *et al.*, 1992). Our assay suggests 25% recombination in *Syn1-cre,Mecp2 ^mCherryStop/y^* and 67% in *Snap25-IRES2-cre,Mecp2 ^mCherryStop/y^* brains in NeuN-low nuclei ( Figure 5). Differences in the mCherry profiles of NeuN-low nuclei emphasised uncertainty. For *Snap25-IRES2-cre,Mecp2 ^mCherryStop/y^* samples, unlike mCherry-high nuclei in the NeuN-high group, the level of mCherry expression in the NeuN-low group did not accurately match the levels in either positive or negative controls, as the profiles were shifted slightly. Flow cytometry alone does not reveal the cellular origin of recombination in NeuN-low nuclei. Specifically, we cannot rule out that a significant subset of glia in *Snap25-IRES2-cre* and *Syn1-cre* mice also express the enzyme.

## Discussion

The immunofluorescence and flow cytometry analysis confirmed the *in situ* hybridisation data of the Allen Institute for Brain Science in showing that only a proportion of neurons (~36%) in the *Syn1-cre* line express Cre. Notably the expression is low in the cerebellum which contains ~60% of all the neurons in the mouse brain ( Erö *et al.*, 2018; Herculano-Houzel *et al.*, 2006) (Blue Brain Cell Atlas: bbp.epfl.ch/nexus/cell-atlas/). In contrast, Cre is active in the majority of neurons (85%) in the *Snap25-IRES2-cre* line. Flow cytometry also identified that both lines express Cre in NeuN-low nuclei which includes non-neuronal cells. The initial characterisation by Zhu and colleagues (2001) of the *Syn1-cre* line concluded that there was no Cre activity in astrocytes as they did not detect Cre reporter expression in astrocytes that stained positively for Glial Fibrillary Acidic Protein (GFAP). Astrocytes make up only 15% of all glial cells in the brain (Blue Brain Cell Atlas: bbp.epfl.ch/nexus/cell-atlas/) thus, further work to characterise the extent of expression of *Snap25-IRES2-cre* in glia would be beneficial, for example by immunostaining of brain sections to assess the degree of colocalization of Cre-reporter and different glial cell markers. A ubiquitously expressed Cre reporter with strong expression in every cell may be more appropriate than the *Mecp2 ^Stop^*, as the low expression of *Mecp2* in glia makes it difficult to reliably distinguish activated from *Stopped* cells. Alternatively, RNA-seq of individual brain nuclei may separate glial from neuronal populations which could be interrogated for MeCP2 expression.

Our study did not investigate the developmental trajectory of *cre* expression in each of these lines. Other groups have addressed this for the *Syn1-cre* line and found that LacZ Cre reporter activation was detectable in the brain, spinal cord and Dorsal Root Ganglion (DRG) from E12.5 onwards ( Zhu *et al*., 2001). Cre activity was detected in differentiated neurons outside the ventricular regions of the brain and spinal cord. These data are consistent with the pattern of endogenous *Syn1* gene expression ( Melloni & DeGennaro, 1994). According to *in situ* hybridisation histochemistry in the rat, the onset of expression of endogenous *Syn1* mRNA coincides with the commitment of neural stem cells to terminal differentiation ( Melloni & DeGennaro, 1994). The onset and pattern of *Snap25-IRES2-cre* expression during mouse development has not been reported. Endogenous *Snap25* can be detected from E12.5 in the mouse brain by RNA-seq analysis ( Cardoso-Moreira *et al*., 2019). SNAP-25 is thought to have a role in axon growth and dendritic arborization as well as the formation and function of synapses ( Grosse *et al.*, 1999; Osen-Sand *et al.*, 1993; Osen-Sand *et al.*, 1996), implying that it is expressed relatively early in neuronal differentiation. SNAP-25 mRNA abundance increases during postnatal brain development, with the largest increase occurring between birth and postnatal day 5, reaching 12-fold higher levels in the adult compared to the embryonic brain ( Oyler *et al.*, 1991). As brain development is a protracted process with different brain regions developing and maturing at different rates the onset of Cre expression in both the *Syn1-cre* and *Snap25-IRES2-cre* lines may follow the developmental time course of each brain region.

Although in both lines Cre expression is largely restricted to the brain, Cre expression in peripheral tissues that have not been analysed so far cannot be ruled out. Despite endogenous Synapsin-1 protein expression being largely restricted to neurons ( De Camilli *et al.*, 1990), very low levels of expression have been reported in small populations of non-neuronal cells including pancreatic β cells, osteoblasts, epithelial cells, and astrocytes in culture (reviewed in Cesca *et al.*, 2010). SNAP-25 protein has been reported to be expressed in some non-neuronal tissues including endocrine cells such as adrenal medullary chromaffin cells and pancreatic β cells, which use similar exocytosis machinery to neurons ( Jacobsson *et al.*, 1994; Roth & Burgoyne, 1994). It remains to be determined whether recombination in the non-neuronal tissues not assayed here might interfere with the interpretation of conditional mutagenesis experiments.

## Conclusion

We compared two mouse lines that drive Cre expression in a broad range of neurons in the brain. Our results confirmed that expression of the *Syn1-cre* transgene is not detectable in more than half of all neurons. In the *Snap25-IRES2-cre* line, Cre was active in most neurons (~85%), but was indetectable in all non-brain tissues that were analysed, including testes. For both these lines, we were unable to rule out the presence of Cre activity in glial cells, as the nuclear fraction with low NeuN could include both neurons and glia

## Data availability

### Underlying data

Zenodo: underlying data for “Comparative analysis of potential broad-spectrum neuronal Cre drivers” https://doi.org/10.5281/zenodo.6783265 ( Paton *et al.,* 2022).

This project contains the following underlying data: 

Figure 1: Raw microscopy image files (.lif) and processed microscopy images (.jpg).

Figure 2: Western blot images for all 3 biological replicates (.png) and quantification data (.xlsx).

Figure 3: Southern blot raw images (.gel), processed images (.jpg), and quantification data (.tif.xlsx).

Figure 4, 5 and supplementary figure 1: Flow cytometry processed data (.png), FlowJo workspace (.wsp) and raw acquisition files (.fcs).

Graph pad files of graphs in figures (.pzfx).

### Extended data

Zenodo: extended data for “Comparative analysis of potential broad-spectrum neuronal Cre drivers” https://doi.org/10.5281/zenodo.6783265 ( Paton *et al.,* 2022).

This project contains the following extended data:

Supplementary figure 1. Flow cytometry gating method used to quantify the proportion of NeuN_high and NeuN_low nuclei which have activated the Cre reporter expression.

### Reporting guidelines

Zenodo: ARRIVE checklist for “Comparative analysis of potential broad-spectrum neuronal Cre drivers” https://doi.org/10.5281/zenodo.6783265 ( Paton *et al.,* 2022).

Data are available under the terms of the Creative Commons Attribution 4.0 International license (CC-BY 4.0).
