# Supplementary material for: Comparative analysis of potential broad-spectrum neuronal Cre drivers
Source: Wellcome Open Res. 2022 Jul 8;7:185. [Version 1] doi: 10.12688/wellcomeopenres.17965.1 (PMC9353198; doi:10.12688/wellcomeopenres.17965.1)
Supplement: Peer review history

# Peer review: Reviewer response for version 1

**DOI:** 10.21956/wellcomeopenres.19911.r51445

**Published:** 2022-08-04

**Authors:** A. Francis Stewart

**Affiliations:** 1 Genomics, Center for Molecular and Cellular Bioengineering, Biotechnology Center, Technische Universität Dresden, Dresden, Germany

The succinct manuscript by Paton *et al.* presents useful evidence showing that the SNAP25-IRES-cre driver is more useful for neuron-specific conditional mutagenesis and transgene activation than the currently preferred Syn1-cre line. This conclusion will be appreciated by many in the neuron specialty field. The manuscript could have been better if the authors had addressed the issue of developmental onset of SNAP25-IRES-cre activity but is nevertheless worthy of publication. Because Syn1-cre is expressed in testes, does this mean that it needs to be introduced through the female line to avoid widespread deletion? Even if this is only an occasional problem, I wonder if the authors could comment on this drawback.

Is the work clearly and accurately presented and does it cite the current literature?

Yes

If applicable, is the statistical analysis and its interpretation appropriate?

Yes

Are all the source data underlying the results available to ensure full reproducibility?

Yes

Is the study design appropriate and is the work technically sound?

Yes

Are the conclusions drawn adequately supported by the results?

Yes

Are sufficient details of methods and analysis provided to allow replication by others?

Yes

Reviewer Expertise:

Epigenetics, genome engineering, conditional mutagenesis.

I confirm that I have read this submission and believe that I have an appropriate level of expertise to confirm that it is of an acceptable scientific standard.

# Peer review: Reviewer response for version 1

**DOI:** 10.21956/wellcomeopenres.19911.r51449

**Published:** 2022-07-20

**Authors:** Gail Mandel

**Affiliations:** 1 Vollum Institute, Oregon Health and Science University, Portland, OR, USA

In this work to determine optimal Cre drivers for mouse brain studies, the authors first tested a commonly cited rat Syn1-Cre driver for excision efficiency in neurons. They confirmed an earlier study that Cre excision was highly mosaic in the *rSyn1-Cre* mice, both within and between brain regions. The authors then did some investigative work and identified a contester for the best pan-neuronal driver, the *Snap25-IRES2-Cre* in the Allen Brain Institute data resource. The authors were in an ideal position to compare these two Cre drivers using two of their established mouse lines for the *Mecp2* gene, one containing a knock-in of mCherry and the other line containing a mCherry tagged *Mecp2* *Stop* allele. By Southern blotting for excision frequency, and flow cytometry for mCherry profiling in high NeuN+ cell populations, the authors found that mice containing the *Snap25-IRES* *Cre* gene showed much higher excision of the tagged *Mecp2* stop allele in neurons, in the whole brain, compared to mice expressing the *rSyn1-Cre* transgene. In low NeuN populations, the authors found varying amounts of Cre excision, precluding conclusions about the underlying cell types. The manuscript is well written and provides a very good review of the relevant literature. Despite the stated limitation of the identity of the non-neuronal cells, the work is valuable for the large number of investigators using the Cre driver approach for manipulating expression in neurons. Many neurological diseases affect neurons across several, if not all, brain regions. In such cases, the corresponding mouse models may benefit from the most widespread Cre expression to identify common underlying mechanisms. Although the authors did not do a comprehensive immunohistochemical analysis of Cre-mediated activation of *rSyn1-Cre* versus *Snap-IRES2-Cre* in the brain, their results indicate a highly significant improvement in Cre neuronal efficiency with the latter driver. I have no major concerns. Below I have listed editorial points for the authors’ consideration.

- Fig. 1. This figure shows the variability of mCherry expression in the *Syn1-Cre* mice among brain regions, but also mosaicism within a brain region, exemplified in the dentate CA3 CA1 and CA1 sectors of the hippocampus. I understand that the authors are concerned with who is the most pan-neuronal, but where the Cre is active within a brain region could have a strong influence on circuitry underlying behaviors, irrespective of the total number of neurons affected. Could they comment on whether ISH data in the Allan Brain Atlas indicates mosaicism of the *Snap25-IRES-Cre* within brain regions? If available, I suspect that the data would show much less mosaicism compared to *rSyn1-Cre*, but if that is the case, stating this finding will further strengthen their point at both levels. Irrespective, authors should indicate either in the figure or legend that dentate CA3 and CA1 are sectors within the hippocampus, and they should say or show where within the cortex and cerebellum the images were taken.
- Fig.2B. Y-axis identifiers should match in the two histograms?
- Fig. 3B. The + sign in B is confusing – can they fit *Mecp2 ^mCherry/y^* over the lanes? 3D. Although it is allowable, showing SD with only two points does not have much meaning. The data could also just be reported in the results section as averages. In any case, if used, the authors need to state the SD values in the figure or legends.
- Figs. 4. The actual density plots for the NeuN data should be shown in this figure, as they did for the mCherry data in the left plots. In that case, the individual histograms could remain in the supplementary data and the histogram in B would remain for quantitation. Why is the intersection of the horizontal lines in plot 2 of Mecp2 ^mCherry/y ^in the middle of a small peak adjacent to a large peak, while in all the other plots the intersection is at the lower points between peaks? How was that intersection determined?  
- Fig. 5. Given all the uncertainties about the constitution of the non-neuronal population and the unexplained profile shifts in the density plots, I would consider moving this figure to the supplement. If not, my ideas about the presentation are the same as in Fig. 4.

Is the work clearly and accurately presented and does it cite the current literature?

Yes

If applicable, is the statistical analysis and its interpretation appropriate?

Partly

Are all the source data underlying the results available to ensure full reproducibility?

Yes

Is the study design appropriate and is the work technically sound?

Yes

Are the conclusions drawn adequately supported by the results?

Yes

Are sufficient details of methods and analysis provided to allow replication by others?

Yes

Reviewer Expertise:

Molecular neurobiology

I confirm that I have read this submission and believe that I have an appropriate level of expertise to confirm that it is of an acceptable scientific standard.
